# The current status and future directions of myxoma virus, a master in immune evasion

**DOI:** 10.1186/1297-9716-42-76

**Published:** 2011-06-09

**Authors:** Bart Spiesschaert, Grant McFadden, Katleen Hermans, Hans Nauwynck, Gerlinde R Van de Walle

**Affiliations:** 1 Department of Virology, Parasitology and Immunology, Faculty of Veterinary Medicine, Ghent University, Salisburylaan 133, B-9820 Merelbeke, Belgium; 2 Department of Molecular Genetics and Microbiology, University of Florida College of Medicine, 1600 SW Archer Rd, P.O. Box 100266, Gainesville, FL 32610, USA; 3 Department of Pathology, Bacteriology and Poultry diseases, Faculty of Veterinary Medicine, Ghent University, Salisburylaan 133, B-9820 Merelbeke, Belgium; 4 Department of Comparative Physiology and Biometrics, Faculty of Veterinary Medicine, Ghent University, Salisburylaan 133, B-9820 Merelbeke, Belgium

## Abstract

Myxoma virus (MYXV) gained importance throughout the twentieth century because of the use of the highly virulent Standard Laboratory Strain (SLS) by the Australian government in the attempt to control the feral Australian population of *Oryctolagus cuniculus *(European rabbit) and the subsequent illegal release of MYXV in Europe. In the European rabbit, MYXV causes a disease with an exceedingly high mortality rate, named myxomatosis, which is passively transmitted by biting arthropod vectors. MYXV still has a great impact on European rabbit populations around the world. In contrast, only a single cutaneous lesion, restricted to the point of inoculation, is seen in its natural long-term host, the South-American *Sylvilagus brasiliensis *and the North-American *S. Bachmani*. Apart from being detrimental for European rabbits, however, MYXV has also become of interest in human medicine in the last two decades for two reasons. Firstly, due to the strong immune suppressing effects of certain MYXV proteins, several secreted virus-encoded immunomodulators (e.g. Serp-1) are being developed to treat systemic inflammatory syndromes such as cardiovascular disease in humans. Secondly, due to the inherent ability of MYXV to infect a broad spectrum of human cancer cells, the live virus is also being developed as an oncolytic virotherapeutic to treat human cancer. In this review, an update will be given on the current status of MYXV in rabbits as well as its potential in human medicine in the twenty-first century.

Table of contents

Abstract

1. The virus

2. History

3. Pathogenesis and disease symptoms

4. Immunomodulatory proteins of MYXV

4.1. MYXV proteins with anti-apoptotic functions

4.1.1. Inhibition of pro-apoptotic molecules

4.1.2. Inhibition by protein-protein interactions by ankyrin repeat viral proteins

4.1.3. Inhibition of apoptosis by enhancing the degradation of cellular proteins

4.1.4. Inhibition of apoptosis by blocking host Protein Kinase R (PKR)

4.2. MYXV proteins interfering with leukocyte chemotaxis

4.3. MYXV serpins that inhibit cellular pro-inflammatory or pro-apoptotic proteases

4.4. MYXV proteins that interfere with leukocyte activation

4.5. MYXV proteins with sequence similarity to HIV proteins

4.6. MYXV proteins with unknown immune function

5. Vaccination strategies against myxomatosis

5.1. Current MYXV vaccines

5.2. Vaccination campaigns to protect European rabbits in the wild

6. Applications of myxoma virus for human medicine

6.1. MYXV proteins as therapeutics for allograft vasculopathy and atherosclerosis

6.2. Applications for MYXV as a live oncolytic virus to treat cancer

7. Discussion and Conclusions

8. List of Abbreviations

References

Author Details

Authors' contributions

Competing interests

Figure Legends

Acknowledgements

## 1. The virus

Myxoma virus (MYXV) is a member of the genus *Leporipoxvirus*, subfamily *Chordopoxvirinae*, belonging to the family of *Poxviridae*. MYXV contains a large linear double stranded deoxyribonucleic acid (DNA) genome consisting of 159 unique viral genes, with diploid terminal inverted repeats (TIRs). Each TIR contains twelve viral genes, nine of which encoding diverse immunomodulating proteins, giving a total of 171 genes [1]. The viral genome is encapsidated within a brick shaped virion, and the complete replication cycle of MYXV takes place exclusively in the cytoplasm of the infected cells from where it expresses a spectrum of host-interactive immunomodulatory proteins [2]. The virus infects only rabbits and European Brown Hares (only occasionally with clinical signs) in the wild, and is nonpathogenic in any tested hosts apart from lagomorphs, but MYXV can replicate in cultured cells from many species, including most human cancer cells which are particularly permissive for MYXV [3,4].

## 2. History

The disease caused by MYXV was seen for the first time in laboratory rabbits in 1896 by Giuseppe Sanarelli in Montevideo, Uruguay [4] Sanarelli was invited by the Uruguayan government to found a new Institute of Hygiene in Montevideo that required the production of antibodies, for which European rabbits were necessary. Following their importation from Europe, these rabbits were housed in an outdoor facility that was open to biting arthropod vectors, such as mosquitoes. Shortly thereafter, the European rabbits were struck down by a highly contagious and lethal disease, which was characterized by multiple lesions and tumors of the skin and conjunctiva. Sanarelli named this new rabbit disease myxomatosis and thought it might be caused by an infectious agent, which was confirmed later on by isolation of the causative poxvirus, called MYXV [4].

The European rabbit evolved in Europe and Eastern Asia, and prior to human intervention was not found in the rest of the world, including Australasia and the Americas. During the colonization of new territories, the European rabbit was often taken along as a source of meat or for sport hunting, and this is what occurred during the colonization of Australia in 1860. Between 1860 and 1930, a growing population of feral European rabbits derived from a small number of initial breeding pairs spread over the entire continent and their numbers rose up to an estimated three billion [5]. Since it was estimated that ten rabbits consumed as much vegetation as a single domesticated sheep, the elimination of rabbits was seen as a very necessary step to economically raise the food and wool production of Australia. Rewards were issued to attract new proposals for reducing the size of the feral rabbit population and one of the people who responded was Louis Pasteur, who recommended using *Pasteurella *cultures that are pathogenic to rabbits. But due to the well-justified skepticism of the Australian government, he was not allowed to release this microbial agent into the wild [4,6].

**Figure 1:**
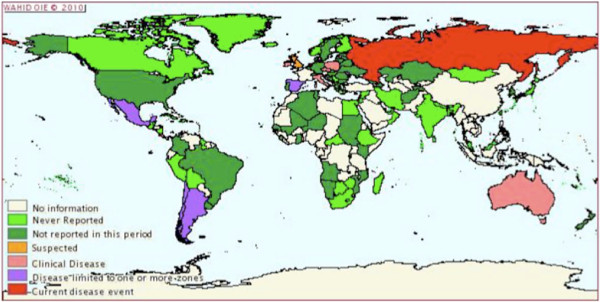
**MYXV worldwide distribution**. The global distribution of MYXV during the period 01/07/2009-31/12/2009 (copied from [152).

Since the original MYXV had a mortality rate of 99.5% in the European rabbit, it was first suggested as a possible means of controlling the Australian feral rabbit population in 1936. Shortly thereafter, the Australian government approved several field release experiments [4,5]. Before the Second World War, two such field tests were conducted, the first of which took place on an island off the South Australian coast and the second in the dry inland outback region. Both experiments showed almost no prolonged spread of the test virus. After the Second World War, MYXV was again tested in a third region in Southern Australia with more seasonal rainfall [6]. Since it was originally thought that MYXV was mostly spread rabbit-to-rabbit by direct contact, the field-tests were conducted mostly in the dry interior of Australia during autumn, winter and spring but without much success in inducing widespread disease in the rabbit population [5]. However, shortly after these field tests when the rainy season ensued, numerous dead MYXV-infected rabbits were found alongside rivers and streams, infected via the seasonally expanded populations of *Culex annulirostris*, a mechanical vector for MYXV. However, it rapidly became clear that almost any biting or sucking arthropod could serve as a vector for the virus, which enabled MYXV to spread over large areas [5,6]. An estimated 400 million rabbits succumbed in as little as one year, and within less than a decade, the feral rabbit population was reduced by 95% [4,6]. In contrast to South America where the virus co-existed with *Sylvilagus sp*. hosts in a relatively nonpathogenic relationship, there was no natural host reservoir species for MYXV present in Australia. Therefore, MYXV was constrained to sustain itself in the same host animals that it was killing with such extreme efficiency. This led to a strong selection for less virulent MYXV strains that killed their European rabbit hosts with less efficiency than the originally introduced high pathogenicity strain of MYXV, enabling more extensive replication and spread of the virus by increasing the survival time of its host [4]. At the same time, the selection pressure exerted by this extremely virulent pathogen caused the host rabbit population to select for the genetically resistant strains that were less susceptible to the lethal disease manifestations of myxomatosis. The co-evolutionary selection pressure reduced the overall mortality rate of MYXV in the field rabbits to less than 30% within seven years after introducing MYXV in Australia. After the introduction of MYXV in Great Britain and France, a similar course of co-evolution between MYXV and its rabbit host was seen [7-9] and at present, MYXV is sustained as a chronic enzootic in rabbit populations in America, Southern Europe, New Zealand and Australia [10] (Figure 1).

## 3. Pathogenesis and disease symptoms

The pathogenesis in immunologically naïve European rabbits after infection with MYXV largely depends on the MYXV strain and the rabbit breed or strain. Aside from naturally occurring MYXV strains, frequently used strains in laboratories are the standard laboratory strain (SLS), the Lausanne strain and the Uriarra (Ur) strain. The SLS and the Lausanne strain, which are both virulent MYXV strains originally isolated in South America, were released in Australia and Europe, respectively [4,7,11]. The Ur strain was isolated from more resistant field rabbits in Australia in 1953, three years after the first release of SLS. Since the Ur strain had already undergone three years of selection to less virulence, it is often used as a model for attenuated MYXV strains [12]. In this review, the pathogenesis of an SLS infection in laboratory rabbits will be described extensively as the canonical example of myxomatosis, whereas the other pathogenesis patterns will be discussed briefly in comparison to this standard model.

**Figure 2:**
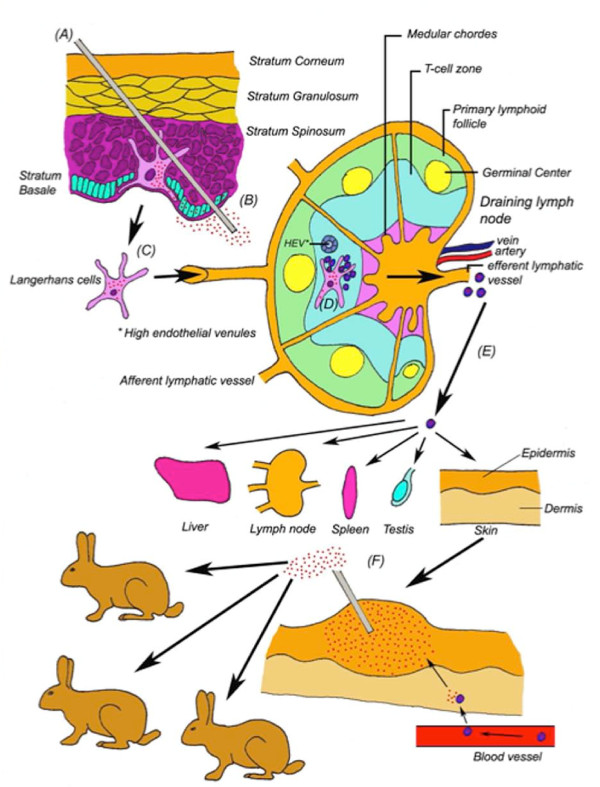
**The pathogenesis of nodular myxomatosis**. At the site of inoculation (A) viral replication occurs first in the peripheral epidermal cells and then in cells deeper in the dermis (B). This initially induces only a mild primary inflammation and mainly attracts neutrophils at early times. Resident Major Histocompatibility Complex-2 (MHC-2) positive cells, such as Langerhans cells, are also infected (C) [2,12,13]. When these cells migrate to the draining lymph node to present viral epitopes in the T-cell zone, they also traffic live virus and T-lymphocytes become infected with MYXV as well (D). This results in wide spread cell death in the T-cell zone of many lymphoid organs and also enables MYXV to spread in a cell-associated manner when these infected T-lymphocytes migrate through the blood and reticuloendothelial circulation. Once in the blood and lymph, MYXV spreads via infected leukocytes to different organs such as the liver, spleen, other distant lymph nodes, testis and epidermis (E). The MYXV replication in these organs induces a strong polymorphonuclear (PMN) cell-influx and in the skin secondary lesions are formed that contain high viral titers, favoring successful spread by mechanical vectors (F) [2,12].

Laboratory rabbits can be infected with MYXV through direct contact with an infected individual, by an arthropod vector, or simply in the laboratory by direct needle inoculation (Figure 2). Through the infection of dendritic cells (DCs) at the primary infection site, and subsequent circulation via the lymphatic and vascular system, MYXV then spreads throughout the body into numerous secondary organs. New myxoma lesions ("myxomes") are formed at multiple secondary sites on the skin where arthropod vectors can acquire high enough titers on their biting organs in order to successfully infect new hosts once they have been serially bitten by the vector (Figure 2) [2,12,13].

This course of disease induces a number of diagnostic clinical symptoms that have been well documented for both the SLS and the Lausanne strains in laboratory rabbits, which are representative for the rabbit population used in either the meat industry or as pets. Essentially all infected immunologically naive laboratory rabbits will develop clinical myxomatosis characterized by a pink color and edema at the point of inoculation, followed by progressive conjunctivitis and serous/mucopurulent secretion from eyes and nose [7,11]. In addition, the infection is accompanied by swelling of the anogenital region and supervening bacterial infections in the respiratory tract as the immune system of the infected host progressively collapses. Animals will usually die 10-14 days post infection (dpi) [14]. Wild Australian rabbits from more resistant populations infected with the SLS strain or laboratory rabbits infected with the Ur strain will display a similar disease course, however usually with a reduced degree of severity, including a delayed onset of clinical symptoms, and a lower overall mortality (Table 1).

**Table 1:** Comparison of symptoms in wild and domestic rabbits infected with different MYXV strains inducing nodular myxomatosis (adapted from [7,11]).

Day pi	Laboratory rabbits			Wild rabbits	
	**SLS**	**Lausanne**	**Uriarra (Ur)**	**SLS**	**Uriarra (Ur)**

1					

2	Pink & edematous inoculation site	Pink/edematous inoculation site		Pink inoculation site	

3			Pink/edematous inoculation site		

4	Conjunctivitis	Conjunctivitis			Pink inoculation site

5		Secondary skin lesions Anogenital edema		Conjunctivitis Anogenital edema	

6	Secondary skin lesions & anogenital edema	Serous eye discharge	Conjunctivitis & Anogenital edema		

7	Respiratory distress	Mucopurulent eye discharge & purple/black inoculation site			Mild conjunctivitis

8		Necrotic inoculation site; Edematous eyelids; Respiratory distress		Secondary skin lesions	

9			Secondary skin lesions		

10	Severe respiratory distress Secondary lesions across the skin ***^#^***	Serous secretion from inoculation site	Respiratory distress	Respiratory distress	Mild anogenital edema; Secondary skin lesions

11		Severe dyspnoea; Secondary lesions across the skin ***^#^***			

12				Lesion regression	

13					

**Day pi**	**Laboratory rabbits**			**Wild rabbits**	

14					

15				Improved breathing; Decreased anogenital edema	Lesion regression

16					

17			Lesion regression		

18					

19					

20			Still recovering	Virtually recovered	

**^#^**: euthanasia of the experimentally infected animals due to too severe clinical symptoms; pi: post inoculation

Apart from the classical nodular form of myxomatosis discussed above, a second less frequent form of myxomatosis exists. This atypical myxomatosis is believed to spread by direct contact, rather then by biting arthropods [15]. The clinical signs of this second form, named amyxomatous myxomatosis, are predominantly respiratory while skin lesions are few and small [16].

## 4. Immunomodulatory proteins of MYXV

The reason for the successful replication of MYXV in rabbits is due to the fact that MYXV encodes a vast array of immunomodulatory proteins (Table 2), some of which are rabbit-specific and others are capable of recognizing immune targets from other hosts, including mice or humans. The gene sequences of these proteins are preferentially located at or near the genomic regions closest to the TIRs of the genome [17] and for many of these proteins, their function has already been investigated in vitro and/or in vivo. For other MYXV proteins, however, their functions are solely predicted by comparing them to analogous related genes from eukaryotic cells or other viruses, such as the vaccinia virus (VACV) [18]. In the present review, the functions of these immunomodulatory proteins will be only briefly discussed but for a more elaborate description of the most important examples, we would like to refer to other recent reviews [19-22]. Moreover, aside from this variety of immunomodulatory proteins, MYXV also encodes genes with important functions in disease pathogenenis, e.g. M127L encodes the DNA repair enzyme photolyase which can promote MYXV survival in the environment [23]. As a description of these proteins is beyond the scope of this review, we would also like to refer for these proteins to other reviews [1,23,24].

**Table 2:** Immunomodulatory proteins of MYXV.

ORF	Protein name	Function	references
M002R/L	M-T2	TNFR homolog; inhibits TNF/TNFR; prevents apoptosis of lymphocytes intracellularly	[27-30]
M11L		Binds with Bak and/or Bax; inhibits apoptosis	[33,34]
M146R		Postulated to bind Bcl-2 proteins and inhibit TLR-mediated innate responses	[1,38]
M005R/L	M-T5	Enhances degradation of CDK inhibitors; prevents checkpoint-induced apoptosis	[42-44]
M150R	MNF	ANK-repeat protein; inhibits NF-κB	[45,48]
M148R		ANK-repeat virulence factor, but no specific function defined to date	[49]
M149R		ANK-repeat virulence factor, but no specific function defined to date	[49]
M153R	MV-LAP	Downregulates cell surface CD4, Fas-CD95 and MHC-1	[13,50,51]
M143		Ubiquitin ligase with a possible anti-apoptotic function	[1]
M004R/L	M-T4	Possible interference with BAP31 in the ER; apoptosis inhibitor	[13,52,53]
M156R		PKR pseudosubstrate and inhibitor	[19,57]
M029L		Predicted inhibitor of PKR via dsRNA binding	[1]
M062R		Host range factor essential for replication in rabbit or human cancer cells; binds SAMD9	[1,61,65]
M063R		Host range factor, essential for replication in rabbit cells	[1,61,62]
M064R		Related to M062 and M064 host range factors but function not yet determined	[1,61]
M001R/L	M-T1	CC-chemokine binding protein	[66,67,153,154]
M104L		Postulated inhibitor of chemokine receptor signaling	[1]
M007R/L	M-T7	Secreted inhibitor of IFN-γ and chemokines; anti-inflammatory activities	[69]
M008R/L	Serp-1	Secreted serpin that inhibits diverse host serine proteases; anti-inflammatory activities	[70,155-157]
M151R	Serp-2	Intracellular serpin that inhibits ICE and granzyme B	[71,73,158]
M152R	Serp-3	Serpin and virulence factor, but no specific host target yet described	[74]
M141R	vCD200	Cell surface virulence factor that inhibits CD200R^+ ^leukocytes (also called vOX-2)	[1,76]
M121R/M122R		Predicted NK regulatory receptor homologs	[1]
M128L	vCD47	Cell surface virulence factor and CD47 homolog	[1,80]
M13L		Pyrin-containing protein that inhibits the inflammasomes and NF-κB signaling	[81,83]
M129R		Predicted HIV gp120 homolog, but function still unknown	[60]
M130R		Predicted HIV Tat homolog; virulence factor	[60,86]
M010L	MGF	Secreted virulence factor, related to TGFalpha and EGF	[89]
M135R		B19R (VACV) homolog, virulence factor, but no specific function or ligand known	[60,90]
M144R		C3L/B5R (VACV) homolog, no confirmed function yet	[60]

### 4.1. MYXV proteins with anti-apoptotic functions

#### 4.1.1. Inhibition of pro-apoptotic molecules

Multicellular organisms use programmed cell death, also designated apoptosis, as an innate defense mechanism to eliminate cells that pose a threat for the rest of the organism, such as virus-infected cells [25,26]. Since apoptosis can have a negative effect on viral replication and spread, many viruses, including MYXV, have developed multiple strategies to inhibit this process. One point of interference is the direct inhibition of key pro-apoptotic molecules.

The MYXV protein *M-T2 (M002R/L) *is a secreted tumor necrosis factor receptor (TNFR) homolog that inhibits apoptosis by binding and subsequently inhibiting rabbit TNF via a conserved ligand-binding domain at the N-terminus of the viral protein [27-29]. A highly conserved viral preligand assembly domain (vPLAD) on the M-T2 protein is responsible for inhibiting intracellular apoptosis, making it the first described viral immunomodulatory protein for which two separate domains with distinct immune inhibiting functions are known [29,30].

*M11L *is a viral protein that localizes to the outer mitochondrial membrane through close association with the mitochondrial peripheral benzodiazepine receptor (PBR) component of the permeabilitytransition (PT) pore. There, M11L prevents the release of mitochondrial cytochrome c induced by staurosporine or protoporphyrin IX (PPIX), which are ligands of PBR [31,32]. Furthermore, M11L is also able to inhibit apoptosis by binding with B-cell lymphoma 2 (BCL2)-antagonist/killer (Bak), which, following pro-apoptotic stimuli, is also able to release cytochrome c and subsequently induce cell death [26,33,34]. Another function for M11L is the ability to interact with activated Bcl-2 associated × protein (Bax), which it inhibits when it is translocated from the cytoplasm to the outer mitochondrial membrane during viral infection [35].

Although not yet demonstrated experimentally, *M146R *is also a potential inhibitor of the pro-apoptotic Bcl-2 family, in the sense that the MYXV protein is closely related to the VACV protein N1L, which has been shown to bind and inhibit Bcl-2 proteins [36,37]. Recently, it was shown that N1L also retains a role in influencing the Toll-like receptor (TLR) signaling pathway [38]. Also, and even more recently, it was found that the VACV ortholog N1L of ectromelia virus (ECTV), the causative agent of mousepox, interferes with host T cell function in vivo, independently of TLR signaling. This was also illustrated by the impairment of in vivo ECTV spread when N1L was deleted [39]. This study provided different results for the virulence factor N1L than those previously predicted in vitro, emphasizing again the importance of studying virulence factors in a natural virus-host system in vivo.

#### 4.1.2. Inhibition by protein-protein interactions by ankyrin repeat viral proteins

Several MYXV proteins contain ankyrin (ANK) repeats and a "Pox protein Repeats of Ankyrin C-terminal" (PRANC) domain (an F-box-like domain), which enable the binding with unique cellular protein targets [40,41]. Through these protein-protein interactions MYXV ANK-repeat proteins act as substrate adaptors that can recruit cellular proteins for ubiquitination and degradation via the 26S proteasome. This is the case for *M-T5 (M005R/L)*, which binds via an adaptor protein called Skp1 with the cellular protein cullin-1 in vitro [42]. Cullin-1 is an E3 ubiquitin ligase, which normally binds to cyclin-dependent kinase (CDK) inhibitors, enzymes that are otherwise degraded in a cell cycle fashion by proteasomes. The binding of M-T5 with Skp1/cullin-1 augments the proteosomal degradation of CDK inhibitors, like p27-Kip, thereby stimulating the progression of the cell cycle past the G_0_/G_1 _checkpoint that could otherwise trigger an apoptosis response when the cell cycle stalls at this checkpoint [42,43]. In vivo experiments have shown that M-T5 is necessary for MYXV to spread secondary immune organs in the infected rabbit host [44], suggesting that M-T5 permits virus spread via infected lymphocytes that traffic to multiple distal immune organs [20].

The myxoma nuclear factor (MNF) *M150R *is crucial for productive viral infection in vivo and is predicted to interfere with apoptosis by inhibiting Nucleus Factor-κB (NF-κB). This prediction is based on the analogy with ANK-repeats and the nuclear location of M150R when compared to cellular NF-κB inhibitors (IκBs) [45]. NF-κB is an important transcription factor that is activated by pro-inflammatory stimuli, subsequently translocating from the cytoplasm to the nucleus where it upregulates host genes critical for immunity, inflammation and apoptosis [46]. IκBs inhibit NF-κB by interfering with the translocation of the active transcription factor dimer to the nucleus and hence, a similar action is predicted for M150R [45,47]. More recently, an alternate possibility of inhibition has been proposed, where M150R directs the ubiquitin ligase complex to new substrate targets, such as NF-κB, for ubiquitination and subsequently degradation [48]. Both *M148R *and *M149R *are also members of the MYXV ANK-repeat family and although the virulence of infection with M148R and M149R deletion mutants was reduced in in vivo experiments, no specific function has yet been determined for these proteins to date [49].

#### 4.1.3. Inhibition of apoptosis by enhancing the degradation of cellular proteins

Apoptosis can also be inhibited by downregulating immune-receptors located on the cell surface, thereby decreasing the chance of recognition by apoptosis-inducing immune cells such as cytotoxic T-lymphocytes [50]. *M153R*, also known as Myxoma Virus-Leukemia-Associated Protein (MV-LAP), is a ubiquitin ligase which achieves the downregulation of a number of immune-receptors at the cell surface, such as major histocompatibility complex 1 (MHC-1), cluster of differentiation (CD)-4 and Fas (CD95), by augmented endocytosis or by decreased recycling to the surface [13,50,51]. For Fas, this downregulation was also accomplished by degradation [50]. The MYXV protein *M143 *is also a ubiquitin ligase, but it remains to be determined whether this viral protein displays a similar anti-apoptotic role as MV-LAP [1].

*M-T4 (M004R/L) *is an anti-apoptotic intracellular virulence factor that has been suggested to function within the endoplasmic reticulum (ER) of infected cells where it binds to calreticulin [13,52,53]. Its anti-apoptotic action, necessary for in vivo spreading throughout the infected rabbit host, is still not understood but it might be explained by a possible interference with the function of BAP31, an ER-localized host protein involved in (i) the egress of MHC-1 and other proteins, (ii) inhibiting apoptosis and (iii) downregulating MHC-1 expression respectively [13,54].

#### 4.1.4. Inhibition of apoptosis by blocking host Protein Kinase R (PKR)

Protein kinase R (PKR) is upregulated by interferon (IFN) and is catalytically activated by double stranded RNA (dsRNA), which can be produced during replication of many classes of viruses, including poxviruses. The activation of PKR causes many downstream anti-viral effects, including apoptosis of the cell, thereby impeding viral spread, and thus many viruses have evolved anti-PKR countermeasures [55]. For example, the VACV K3L protein binds PKR, hereby inhibiting the phosphorylation of the eukaryotic initiation factor 2 alpha (eIF2α), an important regulator of translation initiation [56]. Interestingly, the gene sequence of the protein *M156R *of MYXV shows extensive sequence similarities with VACV K3L and a similar function of M156R was confirmed since M156R protein was shown to be an efficient substrate for PKR in vitro [57]. Indeed, MYXV shows a partial resistance against type-1 IFN in human cells in vitro [58], indicating that MYXV is capable of interfering with IFN signaling in virus-infected cells, even though it is relatively inhibited when the anti-viral state is pre-induced prior to virus infection [59]. It has been shown that MYXV can partially inhibit type-1 IFN signaling by blocking the interferon-induced activation of the Janus kinase Tyk2 in MYXV-infected human cells. The responsible MYXV protein however, is yet to be determined [58]. A conserved region of *M029L*, located in its C-terminal domain sequence, shows strong resemblance with the dsRNA binding region of the VACV gene E3L, which is responsible decreasing the activation of PKR [1,56,60].

In addition, *M062R, M063R *and *M064R *are all orthologs of the VACV host range protein C7L, although only M062R shows similar host range capabilities as C7L as determined by gene swap experiments in the context of a VACV infection [1,61]. *M063R *shows sequence similarity with its C-terminal half a with a glutamate-rich domain of the cellular death-domain associated protein, Daxx, leading to the hypothesis that M063R can inhibit Fas-associated cell death through interfering with the function of Daxx [1,61,62] However, this model remains unproven and it is still not completely understood under which circumstances Daxx functions as either a pro-apoptotic or an anti-apoptotic protein [63,64]. Alternatively, it is hypothesized that M063R functions as a host range gene like M062R, but that it exerts this function through a different pathway [62,65]. Recent evidence suggests that M062R, but not C7L, binds to a specific host anti-viral protein called "sterile alpha motif domain containing protein 9" (SAMD9) implicated in human inflammatory disorders, suggesting that even these closely related viral host range factors may block cellular self-defense pathways by different mechanisms [65].

### 4.2. MYXV proteins interfering with leukocyte chemotaxis

The coordinated recruitment of leukocytes at the site of infection is an important aspect of the early immune response against viruses [66]. Therefore, it is not surprising that poxviruses such as MYXV have also developed multiple ways to interfere with this process. *M-T1 (M001R/L) *is a secreted viral protein that is unnecessary for productive viral replication but it can bind and inhibit chemokines of the CC-subfamily in vitro, thereby inhibiting the directional migration and activation of these cells in vivo [66,67]. In contrast, M-T1 was not able to bind or inhibit chemokines of the CXC subfamily, at least as assessed by chemical crosslinking in vitro, such as CXCL8 (ie IL-8, in humans), and this could explain why neutrophils are the dominant early responder leukocytes at sites of infection in experimentally MYXV-infected laboratory rabbits [12,67].

*M104L *is a small hydrophobic MYXV protein which shares amino acid identity of 42% over a 40-residue region (containing a receptor domain implicated in chemokine receptor signaling) with a subset of the transmembrane domains of the IL-8 receptor analogue ORF74 of Ateline herpesvirus 3, which is believed to inhibit the signaling of a yet to be determined chemokine receptor via heterodimerization, hereby preventing the formation of functional receptor dimers [1]. Another viral MYXV protein, *M-T7 (M007R/L)*, which is an abundantly secreted 37 kDa glycoprotein from MYXV-infected cells, binds rabbit interferon-γ (IFN-γ), hereby inhibiting interferon-γ (IFN-γ) through competitive binding [68]. In addition, it was shown that M-T7 protein is able to bind chemokines of the CXC, CC and C subfamilies in vitro in a pan-species specific fashion, thereby blocking the chemokine-mediated recruiting of leukocytes from many species, including rabbits, mice and humans [69].

### 4.3. MYXV serpins that inhibit cellular pro-inflammatory or pro-apoptotic proteases

Serine protease inhibitors (serpins) are the largest family of cellular protease inhibitors and MYXV encodes three members, each with its own specific functions [1]. *Serp-1 (M008.1R/L) *is a secreted MYXV serpin that is able to bind and inhibit the functions of several human cellular proteases, such as plasmin, urokinase, plasminogene activator and C1s of the complement cascade [70]. However, more research is needed to clarify the interactions of Serp-1 with similar cellular proteases in the rabbit and the implications of such interactions on the course of disease. *Serp-2 (M151R)*, on the other hand, is an intracellular cysteine proteinase that decreases the secretion of bioactive IL-1β by inhibiting the IL-1β-converting enzyme (ICE) in vitro [71]. Serp-2 is also a weak inhibitor of granzyme B, which plays an important role during T cell-induced apoptosis [72,73]. *Serp-3 (M152R) *is the third MYXV protein with a canonical predicted serpin structure, although it has several apparent domain deletions compared to the gene sequences of other serpins [74]. When this *Serp-3 *gene is deleted from MYXV, no secondary skin lesions are observed in rabbits in vivo, despite the fact that no decrease in viral replication nor an increase of apoptosis can be detected in cultured cells [74].

### 4.4. MYXV proteins that interfere with leukocyte activation

MYXV encodes *M141R*, a structural homologue of CD200, which is a cellular membrane-bound immune cell surface protein that is present on a broad range of cells [1,75]. Cellular CD200 functions as a ligand for a receptor (CD200R) which is only present on myeloid cells and is thought to function by transmitting inhibitory signals [75]. It is believed that M141R (also called vCD200 or vOX-2) sends inhibitory signals to CD200R^+ ^DCs or macrophages, hereby decreasing the capacity of antigen presenting cells to activate lymphocytes [76,77]. This theory is supported by some in vivo data, where an increased activation of circulating lymphocytes and macrophages was observed when rabbits were infected with a recombinant MYXV mutant lacking M141R [76].

Two other predicted MYXV proteins, *M121R *and *M122R*, have been proposed to bind with MHC-1 on the cell surface, since they have a similar gene sequence as two natural ligands of MHC-I, namely the natural killer cell group 2 (NKG2) in humans and the lymphocyte 49 (LY-49) receptors in mice [1]. ***M128L (vCD47) ***is another MYXV immunomodulator, encoding a membrane-associated protein that resembles the cellular immune CD47, also known as the integrin associated protein (IAP) [1]. CD47 functions as a stimulatory cell surface ligand for the signal regulatory protein α (SIRPα) on the surface of myeloid cells [78]. CD47, together with SIRPα, form a cell-cell communication system mediating immune cellular functions such as adhesion, mobility, activation and phagocytosis of leukocytes [78,79]. The current hypothesis is that M128L competes with CD47 for binding to SIRPα, hereby disrupting the leukocyte cell functions described above [80].

Pro-inflammatory caspases also play an important role in the immune responses by regulating the activation and secretion of various pro-inflammatory cytokines like IL-1β and IL-18 [81]. These caspases are activated by a multiprotein complex named the inflammasome [82]. *M13L *is a pyrin domain (PYD)-containing MYXV protein that binds with a pyrin-containing component (called ASC) of the inflammasome, thereby inhibiting the activation of IL-1β and IL-18 [81]. In addition, M13L also directly inhibits cellular NF-κB signaling by binding NF-κB1/p105 protein, which regulates the secretion of pro-inflammatory cytokines such as TNF, IL-6 and monocyte chemotactic protein (MCP)-1, thereby giving M13L a dual immune-subversive role by inhibiting both the inflammasome and NF-κB signaling [83]. Finally, *M154R *encodes a protein presenting 50% identity with M2L, a vaccinia virus gene that was also shown to inhibit induction of NF-κB activation [45,84].

### 4.5. MYXV proteins with sequence similarity to HIV proteins

Most genes of MYXV have orthologous family members in other poxviruses, with the exception of M129R and M130R. These two MYXV proteins exhibit a partial sequence similarity with key proteins from another highly immunomodulatory virus, namely human immunodeficiency virus (HIV). The MYXV protein *M129R *partly resembles the V3 loop of the HIV glycoprotein 120 (gp120) (critical for HIV binding with CCR5), but lacks a transmembrane domain and a signal sequence. The gp120 protein is involved in HIV entry through binding with CD4 and CCR5 or CXCR4. These receptors normally fulfill important roles in T-lymphocyte effector functions [85]. It has been hypothesized that M129R may fulfill a similar role as gp120, namely mediating viral entry, but due to the lack of a transmembrane domain, it is not clear yet if M129R is expressed at all on the viral envelope of MYXV [60]. The MYXV protein *M130R *is expressed as a late protein and exhibits some sequence similarity with the glutamine rich region of the HIV protein transactivator (Tat) [60,86]. Tat is partially released from HIV-infected cells, although other analyses also revealed a nuclear location for this protein [87]. Many different functions have been accredited to Tat, including RNA binding, inhibition of T-cell proliferation, induction of apoptosis in T-cells and neurons, inhibition of phagocytosis, decrease of apoptosis in infected cells, interfering with NK-cells, etc. Because of the large variety in functions of Tat, the MYXV protein M130R needs to be tested for all these functions to elucidate if M130R actually shares any functional relationship with HIV Tat [60,88].

### 4.6. MYXV proteins with unknown immune function

Finally, there are also still many MYXV proteins for which no function or sequence similarity with other obvious immunomodulatory counterpart proteins have been found to date. The myxoma growth factor (MGF), also designated *M010L*, is a secreted virulence factor with similarity to transforming growth factor-alpha and epidermal growth factor (including six conserved cysteine residues involved in Epidermal growth factor (EGF) receptor binding), which is necessary for a successful infection in vivo, although the underlying mechanism is still to be determined [89]. Because of a strong sequence similarity with the VACV protein B19R from strain Copenhagen (called B18R in the VACV strain Western Reserve), it was originally thought that *M135R *was a viral scavenger of type-1 IFN, thereby inhibiting this ligand family [60,90-92]. However, this hypothesis has not been validated since all binding and inhibition assays conducted to date, designed to demonstrate that M135R can indeed interact with type-1 IFN, were negative [90]. Nevertheless, M135R remains an important virulence factor for myxomatosis, so more research on the exact function of this protein is warranted.

Finally, the MYXV protein *M144R *shows similarity to the VACV proteins C3L (31% by means of blast search) and B5R (37% by means of alignment analysis) [1,60]. These latter proteins have a complement-binding and structural function respectively, making it tempting to speculate that M144R also possesses these functions, although this still needs to be confirmed [60].

## 5. Vaccination strategies against myxomatosis

### 5.1. Current MYXV vaccines

Prevention of myxomatosis is a matter of great importance for industrial/domestic rabbit farms for laboratory and pet rabbits because of its high prevalence and mortality rate [10]. This prevention can be partly achieved through controlling vectors such as mosquitoes and fleas. However, such sanitary measures are rarely sufficient and therefore, an effective vaccination strategy is desired for successful protection against MYXV [93]. Since a robust cellular immunity is necessary for protection against MYXV, inactivated vaccines have generally proven unsuccessful [93]. Plasmid-based subunit vaccines were also unable to protect rabbits from disease, even though both antigen-specific cell-mediated and humoral immune responses were induced [94]. Therefore, only live vaccines, which can be divided into two groups, are commercially available to date. The first group of vaccines consists of heterologous vaccines based on the closely related but nonpathogenic leporipoxvirus Shope fibroma virus (SFV), due to the close antigenic resemblance of this virus with MYXV [93]. SFV originates from the Eastern cottontail rabbit (*Sylvilagus floridanus*) and provides considerable cross-protection against MYXV without causing disease in European rabbits older than two weeks [2,10]. Possible reasons for this attenuated phenotype of SFV might lie in the partial or complete deletion of several SFV genes found in MYXV, such as those encoding predicted (eg M150R, M139R) or confirmed (eg Serp-1, M135R) virulence factors [95]. Current commercial SFV vaccines are based on the original Shope OA strain, Boerlage strain or closely related strains [93]. The main disadvantage of these vaccines is that they only induce a limited amount of protective immunity that lasts no longer than three months [96,97]. Contradictory findings have been reported as to whether SFV-based vaccines can completely prevent clinical signs of myxomatosis upon infection [98,99]. A possible explanation in this regard could be differences in the adjuvant used or the dosage of vaccine virus used [98]. In addition to adjuvant and dosage of the vaccine virus used, also the vaccination schemes (route, number of injections) could influence the level of protection [99] The second group of vaccines consists of homologous attenuated live vaccines, such as the SG33, Borghi or BTK/RB/84 strains of MYXV [93,100]. SG33 is a homologous vaccine strain, derived from the Lausanne strain of MYXV by serial passages on RK13 rabbit kidney and chicken embryo cells at 33°C [101], during which genomic deletions were introduced [15]. Specifically, a large deletion near the right end of the genome was seen during preliminary analysis [102]. This was later confirmed when the SG33 genome was analyzed [15,45]. These deletions include the loss of important immunomodulatory genes, such as Serp-2, M148R, M149R, M150R, M152R, M153R, M154R and M-T1 [15,45]. In addition, a mutation of the M143 gene was reported in the SG33 strain [15]. The recent analysis of the entire SG33 genome also gave new information concerning the origin of this vaccine strain. It was established that its present sequence composition is the result of field recombination between a wild-type Lausanne strain and a Californian MSD-derived vaccine strain [45]. The Borghi strain is a homologous vaccine strain, derived from the Californian strain MSD of MYXV by serial passages in RK13 cells. The attenuation of the Borghi strain could be due to truncation of Serp-2, M-T4 and/or a mutation in the M121 gene [15]. These homologous MYXV-based vaccines induce a stronger protective immune response than any of the current SFV vaccines and they induce disease protection at least four months after vaccination [93]. The main disadvantage of these vaccines, however, is a reported immunodepression in young rabbits after vaccination. Still, no problems were reported after vaccination with SG33 of young Angora rabbits, which is a breed known to be particularly sensitive to the myxomatosis virus under field conditions [103]. Other side effects reported include lesions of the skin, edema and rash at the injection point, as well as secondary myxoma lesions after vaccination with SG33 [103]. Recently, vaccination with an Uriarra strain deficient for M063R was also evaluated giving long-term results similar to those of heterologous live vaccines [104]. More potent vaccine candidates have been constructed through deletion of one or more virulence genes (e.g. M-T7 or M007L/R, M010L and M011L) in the naturally attenuated MYXV Ur strain [7,12,105]. Vaccination with these vaccines, however, is accompanied by mild clinical symptoms in adult rabbits, thereby making them still too virulent for widespread use as a vaccine. When all three deletions were created simultaneously in the same vaccine, no symptoms were observed in test rabbits but the long-term protection against wild-type MYXV was also limited [7,12,105]. By inserting a VP60 (rabbit hemorrhagic disease (RHD) capsid component that induces a protective antibody response against RHD [106]) construct in the SG33 strain, another potent vaccine strain was created which protected rabbits against both MYXV and RHD [107]. Despite showing promising preliminary results, this recombinant vaccine is still not commercially available [93].

In general, the currently recommended vaccination scheme against myxomatosis consists of a primary vaccination with SFV at three or four weeks of age and a booster vaccination with a homologous MYXV-based vaccine three weeks to two months later, depending if the rabbits are exposed to a high or low infection risk. The rabbits then need to be re-vaccinated every four to six months, depending on the infection risk [93].

### 5.2. Vaccination campaigns to protect European rabbits in the wild

The European rabbit is also an important part of Europe's ecosystems and it is an essential source of food for at least 29 predatory species [108]. MYXV, together with RHD and hunting, is an important reason for the poor condition of some of these indigenous prey populations, such as the Iberian lynx and many raptors [109]. Management strategies such as captivity breeding, restocking, translocations, predator control or habitat management, aimed at raising the number of rabbits for hunting and conservation purposes, have been attempted but they have had very little impact on the overall rabbit population numbers, especially in Mediterranean ecosystems [110]. Therefore, management strategies should include a vaccination program, with adequate procedures [97,110]. However, all of the currently available commercial vaccines are not suitable for vaccination in the wild since they have to be administered individually [93,111]. Therefore, attempts have been made to develop transmissible vaccines that are able to serially spread throughout the rabbit populations in the wild. For this reason, twenty MYXV field strains were isolated from the wild and were evaluated for their virulence and horizontal transmissibility [112]. One of these field strains, isolate 6918, showed appropriate characteristics for safety and immunization, which also remained the case after inserting a VP60 construct for inducing additional immunization against RHD [112-114]. The attenuated phenotype of isolate 6918, in comparison to the Lausanne MYXV strain from which it originated, is possibly caused by frameshift mutations that severely disrupted at least four different viral genes (M009L, M036L, M135R, M148R) [115]. The disruption of M135R and M148R could be important since they are known virulence factors [49,90,115]. The use of such a vaccine, with a successful horizontal transmission capacity, could eventually lead to the protection of a sufficient portion of the rabbit population after capturing, directly vaccinating and releasing a small number of rabbits [112]. Therefore, a field test was authorized on a small island near mainland Europe with a dense rabbit population. After inoculating one fourth of the population, a limited transmission was seen with a seroconversion in 50% of the non-inoculated rabbits [116]. Although promising, the relatively low transmissibility might be an obstacle for immunization in less densely populated areas, so more field tests are needed to fully investigate the potential of this vaccine [117].

Apart from the vaccination itself, the time of vaccination has to be taken into account also in order to maximize the vaccination campaign's effectiveness in areas where myxomatosis is already enzootic. The time of vaccination would be most efficient before the start of the yearly epidemics, since the herd immunity will then be at its lowest and numerous non-immune kits will have been born [117]. Research has shown that there is a two-month window between the birth of susceptible animals and the appearance of a myxomatosis epidemic, which would be the best time for introducing a new MYXV strain in an already endemic area [118]. This, however, could be difficult to implement since the timing of the epidemics varies yearly [117].

## 6. Applications of myxoma virus for human medicine

### 6.1. MYXV proteins as therapeutics for allograft vasculopathy and atherosclerosis (Table 3)

**Table 3:** Clinically adapted MYXV proteins for treating inflammatory diseases.

Gene product	Potential clinical use	References
Serp-1	Secreted serpin that effectively inhibits vascular inflammatory responses and has been tested in human clinical trials to treat acute coronary syndrome.	[122-125]

Serp-2	Viral anti-apoptotic serpin effective in animal models but not yet tested in humans.	[19]

M-T1	Chemokine inhibitor that reduces the allograft rejection and stabilizes plaque formation during artherosclerosis.	[127-129]

M-T7	Chemokine inhibitor that attenuates systemic inflammatory responses and inhibits leukocyte infiltration, thus preventing transplant rejection.	[126-129]

M-T2	TNF/TNFR inhibitor but not yet tested as a potential therapeutic.	[28,30,129]

Transplant vasculopathy is a pathological syndrome that is associated with chronic rejection of organ transplants and it is the leading cause of transplanted organ loss after the first year post-transplant [119]. Its histopathology is characterized by narrowing of the vascular lumen as a result of proliferation of smooth muscle cells and fibroblasts. Furthermore, material of the extracellular matrix is deposited and activated leukocytes infiltrate the vessel wall. There is also a large histopathological resemblance between graft vasculopathy and non-transplant forms of atherosclerosis, due to overlapping pathogenetic pathways [120]. Atherosclerosis can be caused by many factors, including hyperlipidemia, diabetes and hypertension [121]. Numerous proteases are important factors for the development of atherosclerosis, since they help regulate atherosclerosis-related vascular remodeling. An important group of these proteases are the serine proteases since they not only mediate matrix breakdown, but also activate and release diverse pro-inflammatory cytokines and growth factors, thus activating a wide spectrum of leukocyte receptors and promoting increased leukocyte migration into damaged tissues [122]. The ability of purified recombinant *Serp-1 *protein to inhibit several of these serine protease in a species-independent manner raises the possibility that this viral protein could attenuate the vascular inflammatory responses in a wide variety of cardiovascular diseases, such as atherosclerosis, transplantation rejection and injury vasculopathy [122,123]. Indeed, there are potentially numerous applications in human medicine, for example treating other diseases driven by systemic inflammation. Recently, successful human Phase II clinical trials have been reported using purified Serp-1 protein to treat patients with acute coronary syndromes [124,125].

The MYXV protein *Serp-2*, on the other hand, is an intracellular regulator that selectively binds and inhibits mediators of T-cell killing, enabling a generalized reduction of arterial inflammation through a granzyme B/perforin dependent pathway. This may prolong certain specific T-cell functions, allowing subsets of T-cells to provide anti-inflammatory actions or alternatively it may initiate apoptotic responses in disease-causing macrophages and/or smooth muscle cells [122]. Serp-2 protein has not yet been tested in clinical trials [19].

Another important feature of vascular inflammation is the attraction of leukocytes by chemokines to specific sites in the vasculature and their migration through surrounding tissues [121]. The *M-T1 *and *M-T7 *secreted proteins of MYXV, which inhibit in a species-independent fashion a variety of diverse chemokines, block the chemotaxis of leukocytes into these sites of inflammation, subsequently reducing the allograft rejection in a murine aortic allograft model [126-129]. In the case of immune-based joint diseases like rheumatoid arthritis, high quantities of pro-inflammatory cytokines, such as TNF, are found in the synovial fluids and the amount of TNF elevation closely correlates with disease severity. Subsequently, TNF is considered an important target for treatment [129]. The MYXV protein *M-T2 *has high affinity for rabbit TNF, and also can bind and inhibit human TNF-receptors, but more research is still needed to determine if this MYXV protein would have therapeutic potential [28-30,129].

### 6.2. Applications for MYXV as a live oncolytic virus to treat cancer

MYXV is also considered a promising oncolytic virus that is essentially harmless to non-lagomorphs, including mice and humans, but nevertheless is able to selectively infect and kill many diverse human tumor cells. This translational application of MYXV is based on the genetic alterations in intracellular signaling pathways commonly found in cancer cells, which allows the transformed cells to continue dividing without being controlled by normal cellular checkpoints [20,130-136]. On the other hand, these cellular checkpoints also form an important part of the innate self-protection mechanisms against viral infection, and therefore, most transformed cells tend to be more susceptible to many viral infections [134].

The absence of such viral protection mechanisms in cancer cells makes the selective replication possible for at least some oncolytic viruses that normally cannot propagate successfully in human tissues, such as MYXV [134]. More specifically for MYXV, malignant cancer cells in general lose the ability to induce the TNF/IFN-β synergistic anti-viral state, a feature that largely determines the host range of MYXV in primary human cells [130]. In addition, the host range of MYXV in human cancer cells is also influenced by oncogenic activation and tumor suppressor dysfunction [137]. Indeed, MYXV has been shown to be capable of infecting a wide variety of human cancer cell lines in vitro and it has been proven to be a safe and potent oncolytic virus in vivo [132,138,139]. However, in order to be suitable for anti-tumoral therapy in the clinic, MYXV also needs to cause regression of metastatic tumors in immune competent patients [132]. When administered systemically, MYXV was able to decrease murine B16 melanoma lung metastasis in an immune competent mouse model. This regression of tumors was significantly enhanced by the chemotherapeutic rapamycin [140], an effect which was later on shown to be achieved by reconfiguring the internal cell signaling environment of many cancer cells to one which is more optimal for productive virus replication [134]. A more elaborate description of MYXV as an oncolytic virus has recently been reviewed by Liu et al. [19], and the particular exploitation of MYXV to purge cancer cells from human bone marrow transplants is described by Rahman et al. [141].

## 7. Discussion and conclusions

Research on MYXV has been conducted for many years and the interest in this virus is basically two-fold. On the one hand, MYXV is an important pathogen for the European rabbit population and due to economical reasons and ecological consequences, MYXV merits attention for both population control strategies in places (like Australia) where feral rabbit populations have become damaging, as well as vaccination strategies for situations (like commercial rabbit breading operations, or for native rabbits in Europe) where myxomatosis is a detrimental disease. On the other hand, MYXV is also gaining more interest from a therapeutic point of view now that it has been shown to have great potential for treatment of certain inflammatory-based diseases and cancer in humans. Thus, it is of utmost importance to continue to acquire detailed knowledge on the behavior and pathogenesis of this rabbit-specific viral pathogen in both permissive (ie rabbit) and nonpermissive (especially human) hosts.

It has become clear over the years that developing effective vaccines and efficient vaccination strategies for rabbits are not as straightforward as originally thought. The major problems encountered are balancing the need to induce a potent acquired immune response without being accompanied by disease symptoms. Deletions of MYXV virulence genes, but maintaining virus replication fitness, is probably the best approach for a longer lasting immunity, as has been shown by Adams et al. [105]. However, improved potency in terms of the desired immune response was often accompanied by the appearance of some clinical symptoms in vaccinated rabbits, indicating that the level of protection that can be achieved to date with the available vaccines on the market might remain the best approach since these vaccines are of no risk for the rabbit itself.

Another possibility for creating a more potent immune reaction against MYXV could be insertion of important immunogenic genes of MYXV into heterologous vaccine platforms. Such approach has already been reported for canine influenza, equine influenza virus, West Nile virus and bovine viral diarrhea virus, where virulence genes of these viruses were inserted into an equine herpesvirus type 1 (EHV1) vaccine [142-145]. The rationale behind the insertion of MYXV immunogenic genes into an equine pathogen is based on the fact that EHV1 grows extremely well in rabbit cells and hence, rabbit kidney (RK13) are the most commonly used in vitro cell line to grow EHV1 [146,147]. Still, despite this efficient growth in vitro, it needs to be determined whether EHV1 is capable of inducing robust immune responses in rabbits in vivo. Preliminary experiments, however, have shown that rabbits are susceptible for EHV1 infection (Van de Walle et al., unpublished results), indicating that a recombinant EHV1 vector expressing MYXV proteins could prove a novel strategy to efficiently protect rabbits against MYXV. In addition, another possible viral vector vehicle for immunization against MYXV could be exploited with nonpathogenic variants of Vesicular Stomatitis Virus (VSV) since it has shown excellent replication kinetics in the rabbit during experiments for vaccine development [148-150].

Numerous discoveries have been made concerning the immunomodulatory capabilities of MYXV since the sequencing of the full MYXV genome, although the functions of many candidate proteins remain undetermined to date. For some of the latter class of proteins, a putative function had been sometimes ascribed based on sequence similarity with other immunomodulatory viral or cellular proteins, but these conjectures have not always been validated, such as the cases of M063 [1,62] and M135 [90]. This indicates that one needs to be judicious when predicting how a viral protein functions solely based on sequence or structural similarities. Rather, a true biological role can only be assigned after thorough in vitro and in vivo experimentations.

In terms of clinical applications, several of the MYXV immunomodulatory proteins have become promising candidates for treatment of inflammation-based disorders. To date, the first of these virus-derived drugs (Serp-1) has behaved particularly well in humans, with essentially no clinical adverse side effects, in Phase I and II clinical trials. MYXV as an oncolytic virus candidate also shows considerable potential, both against murine cancers in immunocompetent mouse hosts, as well as human cancers xenografted into immunodeficient mice. MYXV appears to be capable of substantially counteracting malignancies derived from many tissues, especially in combination with rapamycin, and the live virus has exhibited essentially no detectable side effects in all murine hosts tested, even mice that are extensively immunocompromised. In the future, insertion of selective anti-cancer therapeutic genes [131] and/or deletion of MYXV virulence genes [151] could further augment its safety against all vertebrate hosts (including rabbits) and therefore improve its clinical suitability. For example, the Interleukin-15 (IL-15) gene has been inserted into the MYXV genome and this recombinant virus remained capable of replicating in vitro to a degree resembling wild MYXV [131]. Very promising results, concerning safety improvement, have also been obtained by deleting the host range genes M063 or M135 since either deletion showed significant attenuation in its natural rabbit host while retaining and even improving the viral oncolytic capabilities compared to wild MYXV [151]. The exploitation of MYXV constructs that are nonpathogenic in all known vertebrate hosts, but still potently oncolytic against human cancers, will hopefully make MYXV an important factor in improving the outcome of treatment for numerous cancers in the future [19].

## 8. List of abbreviations

(ANK): ankyrin; (BCL2): B-cell lymphoma 2; (Bak): (BCL2)-antagonist/killer; (Bax): Bcl-2 associated × protein; (CD): cluster of differentiation; (CDK): cyclin-dependent kinase; (DCs): dendritic cells; (DNA): double stranded deoxyribonucleic acid; (dsRNA): double stranded RNA; (ECTV): ectromelia virus; (ER): endoplasmic reticulum; (EGF): epidermal growth factor; (eIF2α): eukaryotic initiation factor 2 alpha; (gp120): glycoprotein 120; (ICE): IL-1β-converting enzyme; (IAP): integrin associated protein; (IFN): interferon; (IFN-γ): interferon-γ; (IL-15): Interleukin-15; (Ur): Lausanne strain and the Uriarra; (LY-49): lymphocyte 49; (MHC-1): major histocompatibility complex 1; (MCP)-1: monocyte chemotactic protein; (MGF): myxoma growth factor; (MNF): myxoma nuclear factor; (MYXV): Myxoma virus; (MV-LAP): Myxoma Virus-Leukemia-Associated Protein; (NKG2): natural killer cell group 2; (IκBs): NF-κB inhibitors; (NF-κB): Nucleus Factor-Κb; (PBR): peripheral benzodiazepine receptor; (PT): permeabilitytransition; (PRANC): Pox protein Repeats of Ankyrin C-terminal; (PKR): Protein Kinase R; (PPIX): protoporphyrin IX; (PYD): pyrin domain; (RHD): rabbit hemorrhagic disease; (RK): rabbit kidney; (serpins): Serine protease inhibitors; (SFV): Shope fibroma virus; (SIRPα): signal regulatory protein α; (SLS): Standard Laboratory Strain; (SAMD9): sterile alpha motif domain containing protein 9; (TIRs): terminal inverted repeats; (TLR): Toll-like receptor; (Tat): transactivator; (TNFR): tumor necrosis factor receptor; (VACV): vaccinia virus; (VSV): Vesicular Stomatitis Virus; (vPLAD): viral preligand assembly domain.

## Authors' contributions

BS performed an extensive literature study on the subject, analyzed the acquired information and wrote the manuscript; GM and KH both revised the manuscript critically for important intellectual content concerning their own area of expertise; HN was involved in the initial drafting of the manuscript; GVdW participated in its design and coordination, critically revised the manuscript and helped to draft the manuscript. All authors read and approved the final manuscript.

## Competing interests

GM is a co-founder of Viron Therapeutics, which is developing viral proteins as therapeutics. The other authors declare that they have no competing interests.
